# Caregiver Satisfaction with Anxiety Treatment for Autistic Youth: A Mixed Methods Examination

**DOI:** 10.1007/s10803-025-06725-y

**Published:** 2025-02-10

**Authors:** Lesley A. Norris, Jonathan C. Rabner, Marika Marklin, Margaret E. Crane, Kathrin Renschler, Emma Jenkins, Joshua Kemp, Eric A. Storch, Jeffrey J. Wood, Connor M. Kerns, Adam B. Lewin, Brent J. Small, Philip C. Kendall

**Affiliations:** 1 Department of Psychiatry and Human Behavior, Warren Alpert Medical School of Brown University, Providence, RI USA; 2 Kennedy Krieger Institute, Baltimore, MD USA; 3 College of Medicine, Baylor College of Medicine, Houston, TX USA; 4 Departments of Education and Psychiatry, University of California, Los Angeles, CA USA; 5 Department of Psychology, University of British Columbia, Vancouver, BC Canada; 6 Department Pediatrics, University of South Florida, Tampa, FL USA; 7 School of Nursing, University of North Carolina at Chapel Hill, Chapel Hill, NC USA; 8 Department of Psychology, Temple University, Philadelphia, PA USA

**Keywords:** Youth anxiety, Cognitive behavioral therapy, Exposure therapy, Mixed methods, Treatment satisfaction, Systematic inductive thematic analysis

## Abstract

For Cognitive Behavioral Therapy to best meet the specific needs of autistic youth with co-occurring anxiety and to continue to grow as a sustainable treatment option, it is important to incorporate caregiver perspectives and feedback. Data were drawn from a randomized controlled trial and included 148 caregivers of autistic youth (ages 7–13 years, *M* = 9.89, *SD* = 1.79; 23% female; 77.7% White) with co-occurring anxiety disorders randomized to one of two active treatment conditions (*Coping Cat*, *n* = 72, or Behavioral Interventions for Anxiety in Children with Autism [BIACA], *n* = 76). A systematic inductive thematic analysis was used to code open-ended parent responses on the Consumer Satisfaction Questionnaire to identify what caregivers of autistic children with co-occurring anxiety liked most and least about their child’s treatment. Satisfaction with treatment was high (*M* = 64.98, *SD* = 5.48). Caregivers’ most-liked treatment features across treatments included (a) tools and coping skills, (b) therapeutic alliance, (c) caregiver support and involvement, (d) personalized treatment, and (e) treatment efficacy. Least-liked features of treatment and family participation included (a) the commute to the clinic, (b) treatment length, (c) commitment required at home, (d) questionnaires, and (e) scheduling. Treatment responders endorsed therapeutic alliance more frequently. Caregivers in BIACA endorsed caregiver support and involvement at higher rates, in addition to commitment required at home. Caregiver responses indicated a preference for more sessions and highlighted the importance of balancing need for caregiver involvement in treatment while reducing caregiver burden.

Anxiety commonly co-occurs in autistic youth; 39.6% of autistic youth present with at least one anxiety disorder (van Steensel et al., 2011), and parents identify anxiety as the second most common co-occurring mental health concern for autistic youth (Kerns et al., 2020). Anxiety may be even more common in autistic youth than prevalence estimates suggest, given the varied anxiety symptom presentations that have been observed (Kerns et al., 2014). The frequency of anxiety among autistic youth is important, as co-occurring anxiety and autism in youth has been associated with increased symptom severity and functional impairment across a range of domains, including increased self-injury and depressive symptoms (Kerns et al., 2015), poorer social relationships and increased victimization (Ambrose et al., 2021), lower quality of life (van Steensel et al., 2012) and more severe autistic traits, although associations between anxiety and autism correlates are mixed (Vasa et al., 2020). Youth anxiety is also associated with increased stress for caregivers of autistic youth (Kerns et al., 2015). Consequently, intervention for co-occurring anxiety symptoms among autistic youth is critical for improving symptom and functional outcomes for both youth and families.

Cognitive Behavioral Therapy (CBT) is an efficacious intervention for youth anxiety disorders (James et al., 2020; Kendall et al., 2008; Walkup et al., 2008) that has also shown efficacy in improving anxiety among autistic youth (Sharma et al., 2021; Sukhodolsky et al., 2013; Ung et al., 2015; Vasa et al., 2014), particularly with increased parental involvement in treatment (Perihan et al., 2020). For example, in the Treatment of Anxiety in Autism Spectrum Disorder (TAASD) randomized controlled trial (RCT), a standard practice cognitive behavioral intervention for youth anxiety (*Coping Cat*; Kendall & Hedtke, 2006a; Kendall & Hedtke, 2006b) was compared to a cognitive-behavioral intervention personalized for autistic youth with anxiety (the Behavioral Interventions for Anxiety in Children with Autism [BIACA] program). Both CBT conditions outperformed treatment as usual, with additional benefits observed in BIACA on some measures (e.g., primary outcomes, parent-reported internalizing scales; Wood et al., 2020). As treatments like BIACA that target anxiety among autistic youth continue to be developed and implemented, healthcare delivery models suggest that inclusion of key informant perspectives in intervention development and refinement will improve dissemination and sustainable uptake from research to real-world clinical practice (e.g., Rodriguez et al., 2014; Basch et al., 2012). Caregivers’ perspectives are particularly important to integrate into treatment design given the clear positive associations between treatment outcomes for autistic youth with anxiety and increased caregiver involvement (Albaum et al., 2023; Chalfant et al., 2007; Perihan et al., 2020; Puelo & Kendall, 2011; Wood et al., 2020) and the key role caregivers play in managing complex and time-consuming intervention programs for autistic youth (Parker et al., 2020; Ruble & McGrew, 2007).

Qualitative studies clarifying caregiver perspectives of autistic youth treatments that are not focused on anxiety have already been conducted. Themes from this work highlight the importance of caregiver involvement in treatment, although associated challenges have also been documented (Albaum et al., 2023). In one study of caregiver experiences with autistic youth intervention programs not targeting anxiety specifically, caregivers were asked to describe what they liked and disliked about treatments they were currently involved in; themes that emerged from most- and least-liked treatment feature responses centered around treatment effectiveness, relationships with professionals, access to desired treatments, costs (i.e., money, effort, time), concerns about using medications, and caregiver stress (Mackintosh et al., 2012). Examinations of caregiver perspectives focused specifically on anxiety treatment for autistic youth are few. In one study that examined caregiver perspectives regarding a school-based anxiety treatement for autistic adolescents with co-occurring anxiety in Singapore, caregivers reported favorable impressions of the intervention, noting that the concrete structure of the program was useful (Drmic et al., 2017). This is consistent with qualitative findings for youth anxiety treatments not specific to autism, which suggest that caregivers’ most-liked treatment features include structure, coping skills, therapist factors (e.g., alliance, therapist skill), parent involvement, one-on-one time with a therapist, consistency, and personally tailored treatment (Norris et al., 2023).

Additional work is needed to understand caregiver perspectives regarding outpatient evidence-based anxiety psychotherapy treatments for autistic youth. For example, studies of parent-rated therapeutic alliance in CBT for anxiety in autistic youth suggest that the relationship between alliance and outcomes may be lower than in nonautistic samples, underscoring the importance of looking carefully at factors related to treatment satisfaction in this group in particular (Kerns et al., 2018; Klebanoff et al., 2019). To this end, the current study used a mixed method approach (Fetters et al., 2013) within the TAASD study sample to explore what were caregivers' most- and least-liked treatment features of two forms of CBT (*Coping Cat* and BIACA) and to examine differences by treatment responder status and treatment condition.

## Methods

A systematic inductive thematic analysis was applied to open-ended caregiver responses on the Consumer Satisfaction Questionnaire (CSQ; Larsen et al., 1979) to identify themes among caregiver-reported most- and least-liked treatment features. Treatment satisfaction is a widely used indicator of service quality by insurers and providers (Edlund et al., 2003) but infrequently assessed in studies for autistic youth (Albaum et al., 2023): mean satisfaction scores were calculated using concurrently collected quantitative data on the CSQ. To further contextualize qualitative findings, proportional code frequencies for most- and least-liked treatment features were examined separately by (a) treatment responder status and (b) treatment condition. All information is presented in line with the Consolidated criteria for Reporting Qualitative research (COREQ) checklist (Tong et al., 2007).

### Participants

Youth were eligible to participate in the broader TAASD trial if they (1) were age 7–13, (2) met criteria for autism spectrum disorder, (3) met minimum anxiety symptom severity thresholds (PARS ≥ 14; CGI-S ≥ 3) and had anxiety symptoms that were considered the primary mental health problem, (4) received a Verbal Comprenhension IQ score > 70, and (5) were stable on all psychiatric medications. Study exclusion criteria included (1) receiving concurrent therapy targeting anxiety, social skills training or behavior interventions, (2) current clinically significant suicidality or individuals who have engaged in suicidal behaviors within 6 months, (3) nonresponse to an adequate trials of CBT for anxiety within the previous 2 years, (4) lifetime bipolar disorder, schizophrenia or schizoaffective disorder, and (5) initiation of a new psychiatric medication or a dose change on an established psychiatric medication. For further detail, see Kerns et al., 2016.

The current study sample included 148 caregivers of autistic youth with a co-occurring anxiety disorder ages 7–13 years (*M* = 9.89, *SD* = 1.79; 23% female; 18.2% Hispanic or Latino; 77.7% White, 7.4% Other, 5.4% Black or African American, 6.1% Asian, 2.0% American Indian or Alaskan Native, 1.4% missing) recruited across three sites (University of California Los Angeles, University of South Florida, and Temple University) and randomized to one of the two active treatment conditions (*Coping Cat*, *n* = 72, or BIACA, *n* = 76). Approximately half (50.7%) of the sample reported an estimated total household income above $80,000. Modal education level was a standard college degree for both mothers (33.1%) and fathers (33.1%). The majority of caregivers in the sample were married (74.3%). Mothers were the respondents for most CSQs at post-treatment [74.3%; 6.1% father; 1.4% other i.e., great aunt, grandmother/guardian; 18.2% missing].

### Measures

#### Consumer Satisfaction Questionnaire (CSQ; Larsen et al., 1979)

The CSQ is a global 14-item measure of caregivers’ level of satisfaction with the intervention their child received. The CSQ is self-administered and includes 11 quantitative items; sample items include “Overall, my level of satisfaction with quality of the treatment services that I have received is:” and “Would you recommend the treatment(s) that you received to a friend or relative who had a child with an anxiety disorder?” Each item is rated on a 7-point Likert scale, with 1 denoting a negative experience with services and 7 denoting a positive experience. Scores in the current sample ranged from 45 to 74. The CSQ has high internal consistency and has been correlated with therapist estimates of client satisfaction (Larsen et al., 1979). Quantitative sections were followed by three open-ended questions, including two assessing the most- and least-liked treatment features used for the current study (i.e., “*The thing that I liked most/least about the treatment I received was:*”). Cronbach’s α in the current sample was 0.75. The mean number of words used was 11.78 for the most-liked treatment feature (range 1–47) and 7.30 for the least-liked treatment feature (range: 1–43).

#### Anxiety Disorders Interview Schedule for DSM-IV: Parent Versions (Silverman & Albano, 1996) with the Autism Spectrum Addendum (ADIS/ASA; Kerns et al., 2017)

The ADIS/ASA is a semi-structured diagnostic interview used to determine whether youth met Diagnostic and Statistical Manual of Mental Disorders (DSM-IV) criteria for psychiatric disorders in children, with additional clinical guidelines and queries to aid in differential diagnosis of autism and anxiety (Kerns et al., 2017). The ADIS/ASA was administered separately to youth and caregivers by reliable independent evaluators at pre- and post-treatment. This measure has shown reliability and convergent and discriminant validity (Kerns et al., 2017).

#### Clinician Global Impressions-Improvement Scale (CGI-I; Guy, 1976)

Information collected during post-treatment assessments, including the ADIS/ASA, was used to complete the CGI-I, a single item measure of total improvement following treatment ranging from 1 (very much improved) to 7 (very much worse). Youth with ratings of 1 (very much improved) and 2 (much improved) were considered treatment responders (e.g., Walkup et al., 2008), while those with scores of 3 (minimally improved) or greater were non-responders. The CGI-I has shown adequate reliability and validity (Garvey et al., 1999; Perlmutter et al., 1999) and has been positively correlated with improvements in symptom severity and functional impairment (Zaider et al., 2003).

#### Autism Diagnostic Observation Schedule-Second Edition (ADOS-2; Lord et al., 2012)

The ADOS-2 is a gold-standard (Falkmer et al., 2013) semi structured observational assessment administered at screening that measures social, communicative, restrictive and repetitive behaviors associated with autism. The ADOS-2 has demonstrated high interrater reliability, test–retest reliability, and validity (Lord et al., 2012). The ADOS-2 comparison score was used in analyses comparing families with missing and non-missing CSQ scores.

### Treatment Conditions

#### The Behavioral Interventions for Anxiety in Children with Autism (BIACA) program

BIACA included 16 weekly sessions that were 90 min each (45 min with the child and 45 min with caregivers) and two one-hour school consultations focused on improving youth social skills and inclusion. BIACA targeted (1) anxious and avoidant behaviors, (2) social skill differences, (3) restricted and repetitive behaviors, and (4) behavioral problems via in vivo exposure therapy techniques, social skills training, and habit reversal procedures implemented in-session and in the community. BIACA used a modular algorithm-guided format to personalize treatment to each child.

#### Coping Cat

The *Coping Cat* program (Kendall & Hedtke, 2006a, 2006b) included 16 weekly sessions that were 60-min each. Caregiver involvement included (1) regular 15-min check-ins with the caregiver at each session, (2) two caregiver-only sessions in the first half of treatment, and (3) participation in exposure tasks as needed. In the first half of treatment, youth and their caregivers were taught coping skills to address anxiety, which were then practiced in the final half of treatment via behavioral exposure tasks. Similar to BIACA, *Coping Cat* targets youth anxiety using behavioral principles (i.e., modeling, imaginal and in-vivo exposure tasks, role-play, and contingent reinforcement); homework is also used to reinforce and generalize skills learned in session.

### Procedure

Participants completed an initial phone screen, and pretreatment diagnostic and symptom assessment and research questionnaires. Eligible participants were randomized to receive either (1) BIACA, (2) *Coping Cat*, or (3) treatment as usual (TAU). Caregivers included in the current project represented the subset of families randomized to the two active treatments; both conditions resulted in significant positive effects, with BIACA outperforming *Coping Cat* and TAU on some secondary caregiver-reported measures (Wood et al., 2020). After 16-weeks, participants completed the CSQ as part of a larger post-treatment assessment battery and an independent evaluator completed the CGI-I. For further TAASD study details see Kerns et al. (2016) and Wood et al. (2020).

### Data Analysis

Data were stored using SPSS Version 29.0.2.0 and deidentified data were coded in Excel. Themes and codes for most- and least-liked treatment features from the open-ended response question text were developed using a systematic inductive thematic qualitative analysis (Guest et al., 2011). In the first phase of analysis, coders (*N* = 5, LAN, JCR, MM, MEC, KR) read through each open-ended response independently and generated an initial list of response themes using techniques outlined by Ryan and Bernard (2003). Coders then met as a group to discuss their initial lists of themes. At the beginning of this meeting, coders completed an activity to reflect on their positionality and were encouraged to engage in reflexivity throughout the discussion in an effort to reduce bias (Holmes, 2020). The first author used themes that were identified across coders to develop a codebook, which included all core structural components (MacQueen et al., 2008) and is available in supplemental materials. The codebook was used by the first and second author to double code all responses. All responses were coded independently by each coder and any discrepancies in coding were discussed and resolved recursively. As such, interrater reliability estimates are not provided. Quantitative findings were used to expand upon qualitative responses by exploring differences in proportional code frequency (i.e., percentage of caregivers who cited each code) based on responder status (defined using the CGI-I) and treatment condition. Of note, individual participant responses could receive multiple codes (e.g., caregivers may reference both relationship with the therapist and a preference for caregiver involvement as the most-liked treatment feature).

### Positionality and Reflexivity Considerations

All of the present authors identified as White and Non-Hispanic and the majority were male (*n* = 7) without lived experience with autism. All authors described themselves as predominantly cognitive-behavioral or integrative in therapeutic orientation. The subset of authors involved in codebook development/coding had ≥ 1 years of experience delivering exposure-based care to autistic youth with anxiety; degrees at the time of coding included BA (*n* = 2), MA (*n* = 1) and PhD (*n* = 2). Coders were predominantly female (*n* = 4).

## Results

### Missingness

Approximately one third of caregivers did not complete open-ended text responses (*n* = 43 for most-liked treatment feature question; *n* = 47 for least-liked treatment feature question). Within the sample of youth eligible for participation in the study, *n* = 19 did not complete a post-treatment assessment. A series of chi square and t-tests were run to determine whether families with missing CSQ responses or families who did not complete a post-assessment differed from those who completed a post-assessment along demographic measures (youth age, race, and ethnicity; family income), measures of autistic trait levels at baseline (ADOS-2 comparison score), and responder status. There were no significant differences (all *p* > 0.05).

### Overview of Qualitative Findings

Caregivers liked many aspects of the treatments under study, particularly (1) learning new coping skills and tools that could be implemented at home and (2) aspects of their relationship with their therapist. Caregivers appreciated that they were directly involved in treatment, that treatment was personalized to their families, and that they saw improvements in child symptoms and family functioning. Least-liked treatment features often did not focus on treatment specifically, but rather centered around logistical aspects of participation, such as commuting to the clinic, completion of questionnaires, and scheduling. When unliked aspects of treatment were noted, caregivers mentioned a need for more sessions to consolidate gains and described difficulties with the commitment required to implement treatment tasks at home. The themes and frequencies for most-liked and least-liked treatment features are reviewed in more detail below. Code definitions along with representative quotes are presented in Tables 1 and 2 with identifying information redacted.

**Table 1:** Thematic codes, definitions, examples and proportional frequencies for most-liked treatment features

Code name	Abbreviated definition	Quote	Frequency (%)
Tools and coping skills	Specific or general reference to skills and techniques used by the child in treatment	that my child was able to hold discussions about what may be causing his anxiety & fears & actually practicing an activity or situation that he'd usually avoid. Also the relaxation audio has been very helpful as my son is often wound up and very active. (P147)	28.06
Therapeutic alliance	Qualities about the therapist	… I felt like [therapist] really cared about us and liked my kid—that means the world to me (P134)	19.42
Caregiver support and involvement	Importance of family being directly involved in treatment	That I received training, explanation and follow up as well as my child (P533)	17.99
Personalized treatment	Treatment customized to child	The personalization to my son's problems and excintricities (P101)	7.19
Treatment worked	Treatment led to a noticeable change in symptoms	My son improved but my whole life changed. I learned about him, saw things differently, changed my way of parenting. I calm down and in return his anxiety lessened and our lives got better. (we get along now!) (P158)	6.47

* P* participant

**Table 2:** Thematic codes, definitions, examples and proportional frequencies for least-liked treatment features

Code name	Abbreviated definition	Quote	Frequency (%)
Aspects of the commute to the clinic	Parking, traffic, and/or drive time	the drive and parking… (P116)	31.65
Nothing	No changes suggested	There wasn't really anything I disliked about the treatment. (P351)	8.63
Treatment too short	More sessions needed	Too short—I feel that if we continued longer the results would be much more measureable. (P503)	8.63
Commitment required at home	Treatment demanding on families, particularly at home	As a single parent during busy school/work week, It was sometimes difficult to keep up with homework, charts, and rewards at home (P369)	6.47
Questionnaires	Filling out questionnaires	The first and lAst long session with endless questions that asked the same things (P555)	5.76
Scheduling	Inconvenient session times or low availability	I enjoyed the sessions very much but the scheduling was a bit inconvenient (P151)	4.32

* P* participant

### Most Liked Treatment Features

#### Tools and Coping Skills

Caregivers often described tools, techniques, and coping skills provided in treatment as most-liked treatment features. These strategies were often referenced generally (e.g., “*tools to help my child*” [P107]), but also included specific examples such as the FEAR plan, treatment worksheets/workbook, relaxation strategies, and reward charts. Caregivers frequently referenced the importance of homework as a treatment tool, particularly at-home exposure tasks, for the family to practice coping skills learned in therapy in new settings. Caregivers appreciated that these tasks were structured and “*concrete, hands-on… not just talk therapy*” (P307); as one caregiver noted, “*[CHILD] likes instructions so she responded well to the process steps given for her to follow*” (P373).

#### Therapeutic Alliance

Caregivers cited therapeutic alliance among the most-liked treatment features. Caregivers appreciated a feeling of genuine care from clinicians and a sense “*that [the clinician] was really there to help us”* (P310) without judgment. As one caregiver stated, “*to finally have someone understand [CHILD] needs without criticism was a great help to [CHILD]*” (P374). Caregivers also noted the importance of the positive relationship between their child and clinician characterized by a sense of connection and trust (e.g., “*The connection my child felt with his therapist*” [P148] and “*My daughter trusted and felt comfortable and very fond of therapist and program*” [P369]).

#### Caregiver Support and Involvement

Caregivers appreciated that they were an “*active participant*” (P521) and “*100% involved*” (P317) throughout treatment via individual meetings with clinicians and joint child-caregiver meetings. They felt that their involvement in treatment helped them to better understand their child’s behaviors and to learn new strategies to support their child at home. As one caregiver stated, “*it taught me new strategies about how to deal with different problems, and how to make my child independent and improve [h]is autonomy and how to deal with fear, how to reach the reason behind my child reactions (KICK plan)”* (P118). Caregivers noted that they were trained in these strategies in a way that was clear and effective. One caregiver emphasized the importance of experiential learning in this regard, stating, “*In the past I have been told things to do, but never has a doctor or therapist really looked to see what I was doing to perhaps increase these behaviors”* (P342).

#### Personalized Treatment

Caregivers liked that treatment was personalized for their child’s needs [“*The personalization to my son’s problems and eccentricities*” (P116)] and tailored to “*focus on issues specific to my child*” (P170). Caregivers noted that this personalization led to more individualized recommendations and suggestions throughout treatment.

#### Treatment Worked

Caregivers described treatment efficacy as a most-liked treatment feature [(e.g., “*It worked!*” (P535)]. Some caregivers appreciated seeing improvement in their child’s symptoms [“*my child’s decrease in anxiety for the skills we worked on*” (P103)], and others described positive changes in their own approaches to parenting. As one caregiver stated, the treatment “*helped my son and us*” (P372).

### Least Liked Treatment Features

#### Nothing

In line with high satisfaction scores overall, many caregivers did not describe any least-liked treatment features. As one caregiver noted, “*There wasn’t really anything I disliked about the treatment*” (P351).

#### Aspects of the Commute to the Clinic

Caregivers often cited commuting logistics as a least-liked feature of treatment. Some caregivers noted that clinics were located far from their homes [e.g., “*how far it was from my house but was willing to drive it to help my child*” (P345)], which led to caregivers taking their children out of school early and increased anxiety for some youth [“*distance (SITE) being quite a drive may be challenging, at times inc his anxiety level)*” (P145)]. In addition, at certain sites, caregivers often ran into traffic on their way to and from the clinic and had difficulty finding parking upon arrival.

#### Treatment Too Short

Caregivers often described the 16-session treatment as “*too short*” (P313). Caregivers noted that more sessions would have been helpful for further practice utilizing treatment tools and to continue to build upon treatment successes [e.g., “*i feel like it could have been longer cause my child is finally starting to improve and now its over*” (P371)]. Several caregivers felt that if treatment had been longer, symptom improvement would have been more pronounced. As one caregiver stated, “*I feel that if we continued longer the results would be much more measurable*” (P503).

#### Commitment Required at Home

Although caregivers endorsed increased caregiver involvement as a preferred aspect of treatment, some caregivers described difficulty with the time commitment required for them to implement at-home treatment tasks (e.g., coordinating community exposures, implementing reward charts). Caregivers noted that the treatment “*was a big commitment on our part with lots of work involved*” (P539) and that it was often difficult for caregivers to facilitate completion of all tasks assigned in the time between sessions. This was particularly difficult for single-caregiver households.

#### Questionnaires

Questionnaires were often cited as a least-liked treatment feature, although several caregivers noted that they understood their purpose. Caregivers disliked the length of these assessments and the repetitive nature of the questions [e.g., “*The first and last long session with endless questions that asked the same things*” (P555)].

#### Scheduling

Scheduling of sessions was described as a least-liked treatment feature. Per caregivers, the limited availability of treatment slots meant that sessions were at inconvenient times for families and required the child to be taken out of school early, which was not preferred.

### Mixed Method Analyses

Caregivers reported high satisfaction with treatment (*M* = 64.98, *SD* = 5.48, range: 45–74 with a highest possible satisfaction score of 77) and the majority of youth (87.10%) were classified as responders (i.e., CGI ≤ 2) at posttreatment using the CGI-I (“very much improved” *n* = 31; “much improved” *n* = 77; “minimally improved” *n* = 13; “no change” *n* = 2; “very much worse” *n* = 1; missing *n* = 25). There were no significant differences between responders and non-responders along demographic (youth age, race, and ethnicity; family income) or autistic traits (baseline ADOS-2 comparison score) measures.

**Fig. 1:**
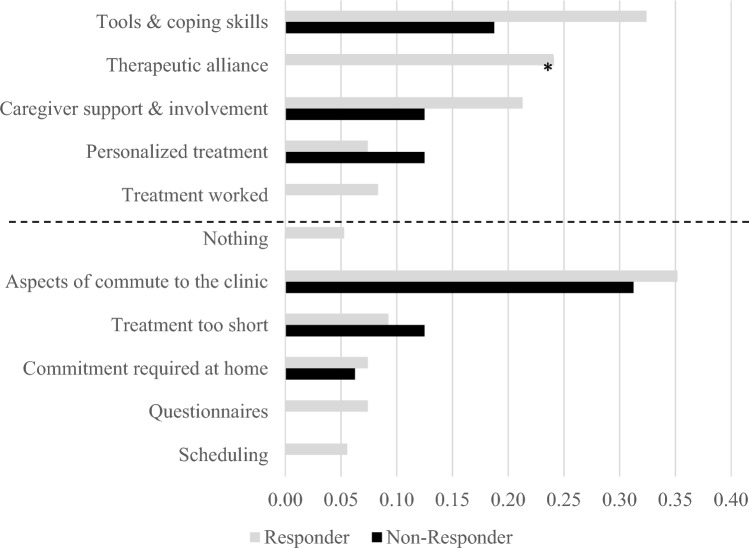
Proportional frequency (%) of most- and least-liked treatment feature themes by responder status. *Indicates a significant difference; most-liked features are above the dashed line, leastliked features are below

Frequencies (in percentage) of themes are presented separately for treatment responders and non-responders based on the CGI-I are in Fig. 1.

**Fig. 2:**
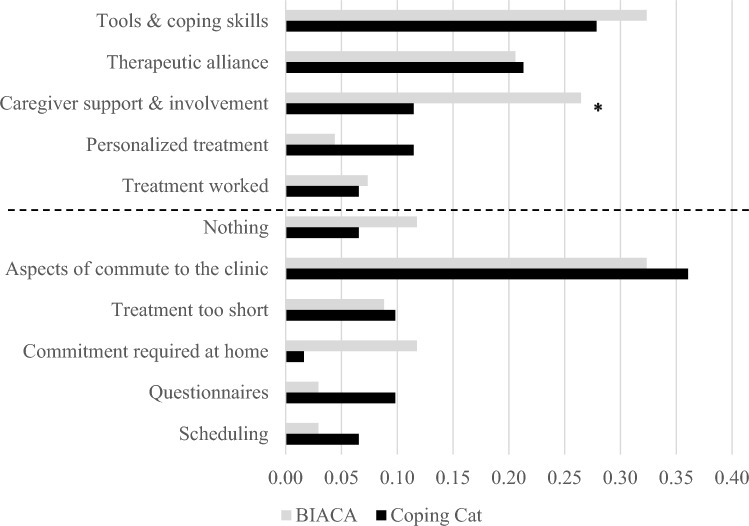
Proportional frequency (%) of most- and least-liked treatment feature themes by treatment condition. *Indicates a significant difference; most-liked features are above the dashed line, leastliked features are below

Frequencies (in percentage) of themes are also presented separately by active treatment condition (*Coping Cat* and BIACA) in Fig. 2.

Chi-square tests compared frequency of most- and least-liked theme endorsement by (a) responder status and (b) active treatment condition. Analyses were conducted in R (R Core Team, 2022), version 3.5.2. For most-liked treatment themes, responders endorsed therapeutic alliance more frequently than non-responders [*χ*^*2*^(1) = 4.87, *p* = 0.03] and more caregivers in the BIACA condition endorsed caregiver support and involvement than those in *Coping Cat* [*χ*^*2*^(1) = 5.13, *p* = 0.02]. For least-liked themes, more caregivers in the BIACA condition endorsed difficulties with commitment required at home [*χ*^*2*^(1) = 5.41, *p* = 0.02]. No other significant differences were found.

## Discussion

Using a systematic inductive thematic analysis to open-ended text data from the Consumer Satisfaction Questionnaire, the present study identified the features of CBT (Coping Cat) and a cognitive-behavioral intervention personalized for autistic children with co-occurring anxiety (BIACA) that were most- and least-liked for caregivers. Consistent with high quantitative satisfaction scores on the CSQ and positive treatment outcomes reported elsewhere (Wood et al., 2020), caregivers cited a range of most-liked treatment features that included both specific parts of the treatment protocol and more general aspects of their involvement in psychotherapy. With regards to specific parts of treatment, caregivers liked the structured provision of tools and coping skills via concrete steps; caregivers noted that more concrete instruction in this way helped facilitate understanding of key concepts. Findings echoed results from previous qualitative work among autistic adolescents with co-occurring anxiety who similarly liked that the anxiety treatment under study was “*a structured program that breaks down an imminent fear into digestible steps. This makes it very easy for both parent and child to understand*” (Drmic et al., 2017, p. 3923) and work among youth with primary anxiety disorders (Norris et al., 2023). When caregivers highlighted specific tools and coping skills, they often noted that homework was helpful, particularly exposure, which further supports previous work showing that exposure is acceptable to families of autistic youth and co-occurring anxiety (Walsh et al., 2018) and that exposure tasks are active ingredients in beneficial gains for youth with anxiety (e.g., Peris et al., 2015, 2017).

In addition, caregivers appreciated the positive relationship between the clinician and their child. The positive association between alliance and outcome has been well-documented in child and adolescent psychotherapy more broadly (*d* = 0.39; Karver et al., 2018; Cummings et al., 2013) and in mental health treatment for autistic youth specifically (Albaum et al., 2023), although other studies hve found a significant relationship between therapist-rated, but not parent-reported, alliance and outcome (e.g., Kerns et al., 2018). Findings from the current project further highlight the importance of the therapeutic relationship in treatment of anxiety in autistic youth. Other most-liked treatment features included treatment efficacy (i.e., that treatment worked) and personalization, which is consistent with previous qualitative work highlighting the importance of treatment effectiveness (Mackintosh et al., 2012) and in line with calls for the development of personalized youth interventions more broadly (e.g., Ng & Weisz, 2016). This is also consistent with the importance of a “flexibility within fidelity” approach to treatment (Kendall, 2021; Kendall & Frank, 2018) in which the manual-based program provides structure and direction but does so with personalized flexibility, which has previously been highlighted as a most-liked feature of *Coping Cat* by caregivers of youth with primary anxiety disorders (Norris et al., 2023). Current findings suggest that caregivers thought that standard CBT was adequately personalized despite being unadapted and not modular.

Caregivers also highlighted several aspects of treatment that were less preferred. One specific update to treatment suggested by caregiver feedback is inclusion of more than sixteen sessions to solidify treatment gains, consistent with caregiver feedback in other samples (Norris et al., 2023) and meta-analytic work suggesting weaker effects in short-term interventions (Perihan et al., 2020). Caregivers otherwise referenced difficulties with logistical aspects of participating in treatment more broadly as least-liked treatment features, consistent with studies of barriers to access of youth psychological treatments more broadly (Reardon et al., 2017). This included commuting to the clinic and scheduling sessions, often because both required youth to miss time in school. Telehealth may address some of these difficulties and is an efficacious treatment option for youth with anxiety and autistic youth (e.g., Conaughton et al., 2017; Rabner et al., 2024), although notably provision of services via telehealth was associated with lower satisfaction with treatment in one study (Ferguson et al., 2022). Other aspects of the family’s participation in a research study were less preferred, particularly completion of redundant questionnaires. Although unique to an RCT setting, findings have implications for introduction of measurement-based care into clinical practice and suggest that, when possible, introduction of less repetitive measures may be preferred for families. Findings with regards to caregiver involvement were somewhat mixed. Many caregivers appreciated being directly involved in their child’s treatment, consistent with previous findings that highlight the importance of increased caregiver involvement in youth autism treatments (Albaum et al., 2023; Chalfant et al., 2007; Perihan et al., 2020; Puelo & Kendall, 2011; Wood et al., 2020). At the same time, others noted that the time commitment of treatment was somewhat burdensome, particularly the time needed to implement at-home treatment tasks. This is in line with previous work noting that a key source of stress among caregivers of autistic youth is managing youth involvement in intervention programs (Parker et al., 2020) that require many hours per week (Ruble & McGrew, 2007). Caregivers themselves offered suggestions for maintaining their involvement while balancing burden, which included more time in between sessions to implement strategies. Notably, concerns about either treatment being invalidating or youth being inappropriately compelled were not raised.

To contextualize qualitative findings, differences in frequencies of theme endorsement by responder status based on the CGI-I and treatment condition were also examined. Caregivers of youth who were classified as responders described liking therapeutic alliance at higher frequencies than families of youth who did not respond, further highlighting the importance of youth-therapist alliance in autistic youth treatments (Albaum et al., 2023; Kerns et al., 2018; Klebanoff et al., 2019). In addition, caregivers randomized to BIACA more frequently described caregiver support and involvement in treatment as a most-liked treatment feature compared to caregivers randomized to *Coping Cat*. Caregivers are involved in *Coping Cat* but are more direct participants in BIACA via longer family/caregiver sessions (45 min versus 15 min) and consistent participation in improving target behaviors and implementing skills (e.g., practicing friendship skills via play-date hosting). At the same time, BIACA families also more frequently cited the commitment required at home to implement treatment as a least-liked treatment feature, suggesting that the increased caregiver involvement, while preferred and associated with increased efficacy (Albaum et al., 2023; Perihan et al., 2020), can be burdensome to some families.

There are several study limitations to consider. First, youth participants did not complete the CSQ and likely had differing perspectives from caregivers regarding their treatment, as previous work suggests higher satisfaction with treatment among caregivers than autistic youth (Klebanoff et al., 2019; Walsh et al., 2018) and discrepancies across informants more broadly (Sharma et al., 2021). Future work should incorporate autistic youth perspectives and include community involvement. Second, families in the current sample were primarily White and Non-Hispanic, consistent with a broader problem of under-representation of Black and Latinx youth in CBT research for autistic youth (Pickard et al., 2019). Caregivers were typically married and of moderate to high socioeconomic status (majority household income > $80,000) with high education levels (majority standard college degree). In addition, mothers more frequently responded to the CSQ open-ended questions. As a result of the lack of representation in the sample, responses may miss the most and least liked treatment features relevant to a more diverse participant group. For example, one caregiver in the current study indicated that increased caregiver involvement was particularly burdensome for single caregiver households; this suggests that increasing representation of diverse family structures could change emergent theme patterns. Limited diversity was also reflected in the coding team, particularly in regards to racial/ethnic, clinical training backgrounds, and lived experience. This represents an area of potential bias, although efforts were made to reduce intrusion of bias throughout coding via regular reflections on positionality and reflexivity. In addition, qualitative data was drawn from open-ended text responses on a measure that included many close-ended questions and, as such, there was no opportunity to expand upon caregiver responses. Future work should be conducted using in-depth interviews or focus groups to facilitate richer responses to the question of what families do and do not like about their treatment.

Overall, study results highlight the importance of assessing key informant perspectives of youth interventions. Findings suggest that caregivers liked that anxiety interventions for autistic youth included provision of tools and coping skills, caregiver support and involvement, and personalization. Caregivers also liked the therapeutic alliance and that treatment was efficacious. When caregivers described least-liked treatment features, they cited aspects of commute to the clinic, completion of study questionnaires, the commitment required at home, and scheduling. They also noted that they would prefer more treatment sessions. Results suggest several future directions for further tailoring of youth anxiety interventions to optimize outcomes for autistic youth, including provision of more sessions and continued balance emphasizing caregiver involvement while reducing caregiver burden, although it is important to note that autistic perspectives were not included in the present work. Future work should continue to center caregiver, patient, and other key informant perspectives voices to inform potential updates to anxiety interventions for autistic youth.
